# Spleen-preserving pancreatectomy with removal of splenic vessels: impact on splenic parenchyma ?

**DOI:** 10.1186/s12893-023-02133-0

**Published:** 2023-08-21

**Authors:** Coralie Lete, Martin Brichard, Maria Luisa Rosa, Mike Salavracos, Catherine Hubert, Benoit Navez, Jean Closset, Martina Pezzullo, Julie Navez

**Affiliations:** 1 Medico-Surgical Department of Gastroenterology, Hepatopancreatology and Digestive Oncology Hôpital Erasme, Hôpital Universitaire de Bruxelles (HUB), Route de Lennik 808, Brussels, 1070 Belgium; 2 Hepato-Biliary and Pancreatic Surgery Unit, Department of Abdominal Surgery and Transplantation, Cliniques Universitaires Saint-Luc, Université Catholique de Louvain (UCL), Avenue Hippocrate 10, Brussels, 1200 Belgium; 3 Department of Radiology, Hôpital Erasme, Hôpital Universitaire de Bruxelles (HUB), Université Libre de Bruxelles (ULB), Route de Lennik 808, Brussels, 1070 Belgium; 4 Department of Radiology, Cliniques Universitaires Saint-Luc, Université Catholique de Louvain (UCL), Avenue Hippocrate 10, Brussels, 1200 Belgium; 5 Surgiprint 3D Intelligence, Louvain-La-Neuve, 1348 Belgium

**Keywords:** Distal pancreatectomy, Spleen preservation, Splenic vessels, Splenic ischemia, Splenic volume

## Abstract

**Background:**

While outcomes after spleen-preserving distal pancreatectomy (SP-DP) have been widely reported, impacts on splenic parenchyma have not been well studied. This study aimed to compare postoperative outcomes, particularly spleen-related outcomes, by assessing splenic imaging after SP-DP with or without splenic vessels removal.

**Methods:**

Data for all patients who underwent SP-DP with splenic vessels removal (Warshaw technique, WDP) or preservation (Kimura technique, KDP) between 2010 and 2022 in two tertiary centres were retrospectively analysed. Splenic ischemia and volume at early/late imaging and postoperative outcomes were reviewed.

**Results:**

Eighty-seven patients were included, 51 in the WDP and 36 in the KDP groups. Median Charlson’s Comorbidity Index was significantly higher in the WDP group compared with the KDP group. Postoperative morbidity was similar between groups. There was more splenic ischemia at early imaging in the WDP group compared to the KDP group (55% vs. 14%, p = 0.018), especially severe ischemia (23% vs. 0%). Partial splenic atrophy was observed in 29% and 0% in the WDP and KDP groups, respectively (p = 0.002); no complete splenic atrophy was observed. Platelet levels at POD 1, 2 and 6 were significantly higher in the WDP group compared to KDP group. At univariate analysis, age, Charlson Comorbidity Index, platelet levels at POD 6, and early splenic infarction were prognostic factors for development of splenic atrophy. No episodes of overwhelming post-splenectomy infection or secondary splenectomy were recorded after a median follow-up of 9 and 11 months in the WDP and KDP groups, respectively.

**Conclusions:**

Splenic ischemia appeared in one-half of patients undergoing SP-DP with splenic vessels removal at early imaging, and partial splenic atrophy in almost 30% at late imaging, without clinical impact or complete splenic atrophy. Age, Charlson Comorbidity Index, platelet levels at POD 6, and early splenic infarction could help to predict the occurrence of splenic atrophy.

## Background

Distal pancreatectomy (DP) is the reference treatment for tumours of the tail and body of the pancreas. Spleen preservation can be performed even when removal of the splenic vessels is necessary. Warshaw described the feasibility of spleen-preserving DP (SP-DP) with splenic vessels resection in 1988 in which the blood supply of the spleen was ensured by the short gastric vessels and the left gastro-epiploic artery [1]. The risk of this procedure is mainly splenic infarction, with a reported rate of splenectomy of 2-5% secondary to spleen necrosis, and asymptomatic perigastric varices due to left-sided portal hypertension [2]. Usually performed for benign or premalignant lesions, SP-DP has been proposed for malignant lesions in the pancreatic body, with lymphadenectomy being performed thanks to splenic vessels resection according to the Warshaw technique [3–6]. Indeed an absence of spleen invasion or splenic hilar lymph nodes (station 10) involvement was observed at pathological analysis of all body tumours compared with tail tumours which present a low risk of splenic parenchyma or lymph nodes involvement. SP-DP has been also described without splenic vessels removal, as reported by Kimura, although it can sometimes be technically challenging due to the close relationship between the splenic vessels and the pancreatic parenchyma [7]. The advantages of spleen preservation during DP include fewer infectious complications, less intraoperative blood loss, a lower overall morbidity rate, and fewer subphrenic abscesses compared to DP with splenectomy [8].

The spleen is a lymphoid organ that plays an important role in the immune system with regard to the storage of blood cells, phagocytosis of encapsulated bacteria and immunoglobulin production. After splenectomy, the risk of infectious complications and overwhelming infections is increased, as well as the risk of thrombocytosis and hypercoagulability [9, 10]. In case of severe splenic ischemia after SP-DP, splenic function can potentially be altered.

While postoperative outcomes after SP-DP have been widely reported in the literature, either with or without splenic vessels removal, the impacts on splenic parenchyma and splenic function have been poorly studied. The aim of this study was to compare postoperative outcome, particularly spleen-related outcomes, by evaluating splenic imaging in the short- and mid-term follow-up after SP-DP with splenic vessels removal or preservation.

## Methods

This retrospective, observational, non-interventional study of patients undergoing SP-DP for pancreatic benign or (pre-)malignant lesions in two tertiary centres, was approved by the Institutional Review Board (reference P2022/325), and was performed in accordance with the Declaration of Helsinki. Written informed consent was waived by the Ethics Committee at Erasme Hospital. The datasets used and analysed during the study are available from the corresponding author upon reasonable request.

All adult patients who underwent SP-DP (laparoscopic or open) between 2010 and 2022 were included in the analysis, excluding patients with total pancreatectomy. The choice of SP-DP technique (with or without splenic vessels removal) depended on the surgical indication (Warshaw DP [WDP] in cases of malignancy for lymphadenectomy) and, in cases of benign or premalignant lesions, was left to the surgeon’s discretion depending on the anatomical relationship between the tumour and the splenic vessels, and the technical difficulty of preserving the splenic vessels.

### Surgical procedures

SP-DP with removal of splenic vessels was performed according to Warshaw technique (WDP) [1]. The splenic vein and artery were ligated medially at the pancreatic transection level and laterally at the splenic hilar. Splenic vessels were removed by performing retropancreatic lymphadenectomy. SD-DP with splenic vessels preservation was performed by dissecting small pancreatic vascular branches from splenic vein and artery which were carefully preserved, as described by Kimura (KDP) [7]. Short gastric vessels were preserved in both techniques. The transection planes differed according to tumour location and surgical indication. For adenocarcinoma, pancreatic transection was at the isthmus. For benign and pre-malignant tumours, pancreatic transection was at the right side of the lesion with a sufficient pancreatic margin.

### Endpoints

The primary endpoint was postoperative splenic imaging, including ischemia on early imaging and volume on late imaging. The secondary endpoints included postoperative complications, peripancreatic collection, perigastric varices, and serum levels of haematological and inflammatory parameters (haemoglobin, platelets, leucocytes and C-reactive protein [CRP]).

### Perioperative outcomes

Patient comorbidities were evaluated according to Charlson’s Comorbidity Index [11]. Postoperative morbidity was defined as any complication until discharge and readmission within 90 days after surgery, and was graded according to the Clavien-Dindo classification [12]. Clavien-Dindo grade ≥ 3 events were considered to be severe complications. To assess all complications that occurred in a single patient, the Comprehensive Complication Index (CCI) was calculated via the CCI calculator available at the CCI website (https://www.cci-calculator.com/cciCalculator) [13]. Postoperative mortality was defined as any death occurring before postoperative day 90. Postoperative pancreatic fistula was classified according to the International Study Group of Pancreatic Surgery [14].

Postoperative follow-up consisted of clinical and biochemical evaluation during the first week. Outpatient follow-up was performed variably depending on surgical indication and on the discretion of the specialist practitioner from the referring institution, in cases of referral of patients for surgery, after 1 to 6 months and after 12 months with routine blood test and abdominal imaging.

### Splenic radiological parameters

Splenic volume and enhancement analyses were performed on preoperative and postoperative contrast-enhanced CT or magnetic resonance imaging (MRI), with a preference of CT over MRI if both were available. Preoperative imaging was the imaging done closest before the pancreatic surgery. Postoperative imaging was performed after 1, 3, 6, and/or 12 months: those at 1 and 3 months were considered to be “early” imaging (with a preference for the 1-month image if both were available), and those at 6 and 12 months were considered to be “late” imaging (with a preference for the 6-month image if both were available).

The presence of a splenic infarction was determined by review of all available CT scans and was graded as follows : grade 0 = 0%; grade 1 = 1–25%; grade 2 = 26–50%; grade 3 = > 50%. Splenic volume (in mL) was measured using semi-automated software. Splenic volume ratio (SVR) (postoperative splenic volume/preoperative splenic volume) was calculated to assess any change between preoperative and postoperative splenic volume. Splenic atrophy was considered when the splenic ratio was ≤ 0.70. Perigastric varices were defined as tortuous vascular structures larger than 5 mm along the gastric wall. All imaging studies were reviewed by experienced radiologists at each institution.

### Haematological parameters

Postoperative laboratory tests studied included serum haemoglobin levels, leucocyte count, platelet count, and CRP levels, performed preoperatively, at postoperative day (POD) 1, then approximately every two days during clinical evolution, and after 30 days (+/- 5 days). Samples harvested on POD 3 were pooled with POD 2 samples when absent, those on POD 5 were pooled with POD 4 samples when absent, and those on POD 7 were grouped with POD 6 samples when absent.

### Statistical analysis

Mean and standard deviation are reported for normally-distributed continuous variables. Median and interquartile range are reported for asymmetric distributions. The normality of distributions was assessed with graphic representations. Means were compared between the groups with Student’s t-test. Asymmetric distributions were compared with the Mann-Whitney-Wilcoxon test. Absolute and relative frequencies are reported for categorical variables, which were compared with the chi-squared test or Fisher’s exact test. A univariate binary logistic regression was performed to assess the association of different factors with splenic atrophy. Statistically significant variables were entered into a multivariate model. An automatic model selection based on the Akaike information criterion was performed to choose the final model. Two-sided p < 0.05 was considered statistically significant. Analyses were performed on SAS 9.4.

## Results

### Patient characteristics

A total of 87 patients who underwent SP-DP were included in the study, 51 were treated according to the WDP technique (splenic vessels removal) and 36 were treated according to the KDP technique (splenic vessels preservation). One patient initially planned for KDP procedure had splenic artery ligation due to technical difficulties, and was not included in the statistical analysis. Patient demographics and surgical indications are detailed in Table 1. The characteristics of included patients were similar with regard to age, sex ratio, and body mass index between the WDP and KDP groups. The median Charlson’s Comorbidity Index was higher (p = 0.003) and the surgical indication tended to be more frequently malignant (p = 0.067) in the WDP group compared to the KDP group.

**Table 1:** Patient characteristics

	WDP group (n = 51)	KDP group (n = 36)	p value
Age, y (median ± IQR)	59 (± 17)	51 (± 14)	0.146
Centre, n (%) - Cliniques Saint-Luc - Hôpital Erasme	27 (53%) 24 (47%)	22 (58%) 16 (42%)	0.618
Sex ratio (m/f)	0.88 (24/27)	0.57 (13/23)	0.381
Body mass index, kg/m^2^ (median ± IQR)	25.4 (± 5.0)	26.5 (± 3.6)	0.510
Charlson comorbidity index (median ± IQR)	4 (± 3)	2 (± 2)	0.003
Indication for surgery, n (%) *Malignant* - Adenocarcinoma - Neuroendocrine tumour - Renal cancer metastasis - Other *Benign* - IPMN - Mucinous cystic tumour - Serous cystic tumour - Solid pseudopapillary tumour - Other	30 (59%) 16 13 1 0 21 (41%) 6 4 1 4 6	14 (39%) 0 12 1 1 22 (61%) 6 5 3 2 6	0.023

IPMN, Intraductal Papillary Mucinous Neoplasm; KDP, Kimura distal pancreatectomy; WDP, Warshaw distal pancreatectomy

### Intraoperative and postoperative outcomes

Most procedures were performed by laparoscopy: 82% and 78% in the WDP and KDP groups, respectively (Table 2). One patient from the KDP group had a pancreatico-jejunal anastomosis on the pancreatic remnant. Operative times and intraoperative blood loss were not significantly different between the groups.

**Table 2:** Intraoperative and postoperative outcomes

	WDP group (n = 51)	KDP group (n = 36)	p value
Surgical approach, n (%) - Laparoscopy - Laparotomy - Lap. converted	42 (82%) 4 (8%) 5 (10%)	28 (78%) 8 (22%) 0	0.034
Operative time, min (median ± IQR)	268 (± 74)	219 (± 63)	0.063
Blood loss, mL (median, Q1-Q3)	200 (0–500)	50 (0–313)	0.342
Severe complication (Clavien-Dindo > 2), n (%)	8 (16%)	5 (14%)	0.817
Death, n (%)	0	0	-
Comprehensive Complication Index (CCI) (median, Q1-Q3)	8.7 (8.7–22.6)	8.7 (8.7–22.6)	0.934
Clinically relevant pancreatic fistula, n (%) - Grade B - Grade C	6 (12%) 6 0	7 (19%) 5 2	0.322
Delayed gastric emptying, n (%)	2 (4%)	1 (3%)	0.773
Haemorrhage, n (%)	2 (4%)	1 (3%)	0.773
Sepsis, n (%)	6 (12%)	3 (8%)	0.605
Surgical reintervention, n (%)	1 (2%)	2 (6%)	0.365
Postoperative hospital stay, days (median, Q1-Q3)	8 (7–8)	8 (7–9)	0.288
Readmission within 3 months, n (%)	10 (20%)	6 (17%)	0.727

KDP, Kimura distal pancreatectomy; WDP, Warshaw distal pancreatectomy

Postoperative morbidity was similar between the two groups, according to the median CCI (p = 0.934) and the rate of severe postoperative complications (p = 0.817) (Table 2). There were no postoperative deaths. No differences were observed in the rates of different types of complications related to pancreatic surgery, including pancreatic fistula, delayed gastric emptying, or haemorrhage. Three patients required surgical reintervention for the following reasons: intraabdominal bleeding (n = 1 in the WDP group, n = 1 in the KDP group), and mis-drained pancreatic fistula (n = 1 from the KDP group). The median postoperative hospital stay was 8 days in both groups (p = 0.288), with similar readmission rates within 3 months (20% and 17% in the WDP and KDP groups respectively, p = 0.727).

### Spleen-related outcomes

Early and late postoperative imaging could be evaluated in 61 (70%) and 64 (74%) patients, respectively (Table 3). In the WDP group, there was an increase in postoperative splenic ischemia at early imaging compared to the KDP group (55% vs. 14% respectively, p = 0.018), especially severe ischemia (> 50% of parenchyma, 23% vs. 0%), and, surprisingly, 3 patients from the KDP group were observed with a grade 1 splenic ischemia. There were significantly more peripancreatic collections in the KDP group (p = 0.039) but these tended to be smaller in size compared to the WDP group (p = 0.055). In 6 patients (4 in the WDP group and 2 in the KDP group), collections required endoscopic ultrasound-guided drainage using double pigtails. Four patients developed a persistent pancreatic fistula which required either endoscopic retrograde pancreatic drainage using a plastic pancreatic prosthesis (n = 2, from the KDP group), or endoscopic retrograde pancreatography associated with percutaneous radiological drainage (one from each group).

**Table 3:** Spleen-related postoperative outcomes

	WDP group		KDP group		p-value	
**Early postoperative imaging** (70%)	40/51 (78%)		21/36 (58%)			
Delay surgery / early imaging, days (median, Q1 – Q3)	72 (28–114)		34 (28–86)		0.196	
Early splenic infarction - Grade 0 - Grade 1 - Grade 2 - Grade 3	19 (48%) 9 (23%) 3 (9%) 9 (23%)		18 (86%) 3 (14%) 0 0		0.018	
Early peripancreatic collection, n (%)	24 (60%)		18 (86%)		0.039	
Peripancreatic collection size, mm (median ± IQR)	61 (± 29)		43 (± 30)		0.055	
**Late postoperative imaging (**74%)	35/51 (69%)		29/36 (81%)			
Delay surgery / late imaging, months (median ± IQR)	7 (± 4)		6 (± 3)		0.429	
Late perigastric varices, n (%)	23 (66%)		20 (69%)		0.783	
Splenic volume ratio, median (± IQR)	0.92 (± 0.53)		1.21 (± 0.41)		0.054	
Splenic atrophy (ratio < 0.70), n (%)	10 (29%)		0		0.002	
**Long-term (at last follow-up) spleen-related complications**						
Gastric bleeding from perigastric varices		0		0		
Splenectomy	0		0			
Overwhelming post splenectomy infection	0		0			

KDP, Kimura distal pancreatectomy; WDP, Warshaw distal pancreatectomy

At late postoperative imaging, perigastric varices were identified in approximately 66% and 69% of patients in the WDP and KDP groups, respectively (p = 0.783). All patients from the KDP group had patent splenic vessels. The median splenic volume ratio was smaller in the WDP group compared to the KDP group (p = 0.054), and partial splenic atrophy was observed in 10 patients from the WDP group (29% vs. 0%, p = 0.002). The smallest splenic volume ratio observed in the WDP group reached 0.41; no complete splenic atrophy was observed. In these patients, 8 had early postoperative imaging, on which splenic ischemia was observed in 7 (> 75% of parenchyma in 4). There were no recorded episodes of overwhelming post-splenectomy infection or bleeding gastric varices in either group after a median follow-up of 9 [3–12] and 11 [6–13] months in the WDP and KDP groups, respectively, and no patients experienced secondary splenectomy during the study period.

The patient initially planned for a KDP procedure who had splenic artery ligation developed grade 1 splenic ischemia at early imaging (1 month), and a splenic volume ratio of 1.31 after 6 months, without any perigastric varices.

### Haematologic parameters

**Fig. 1:**
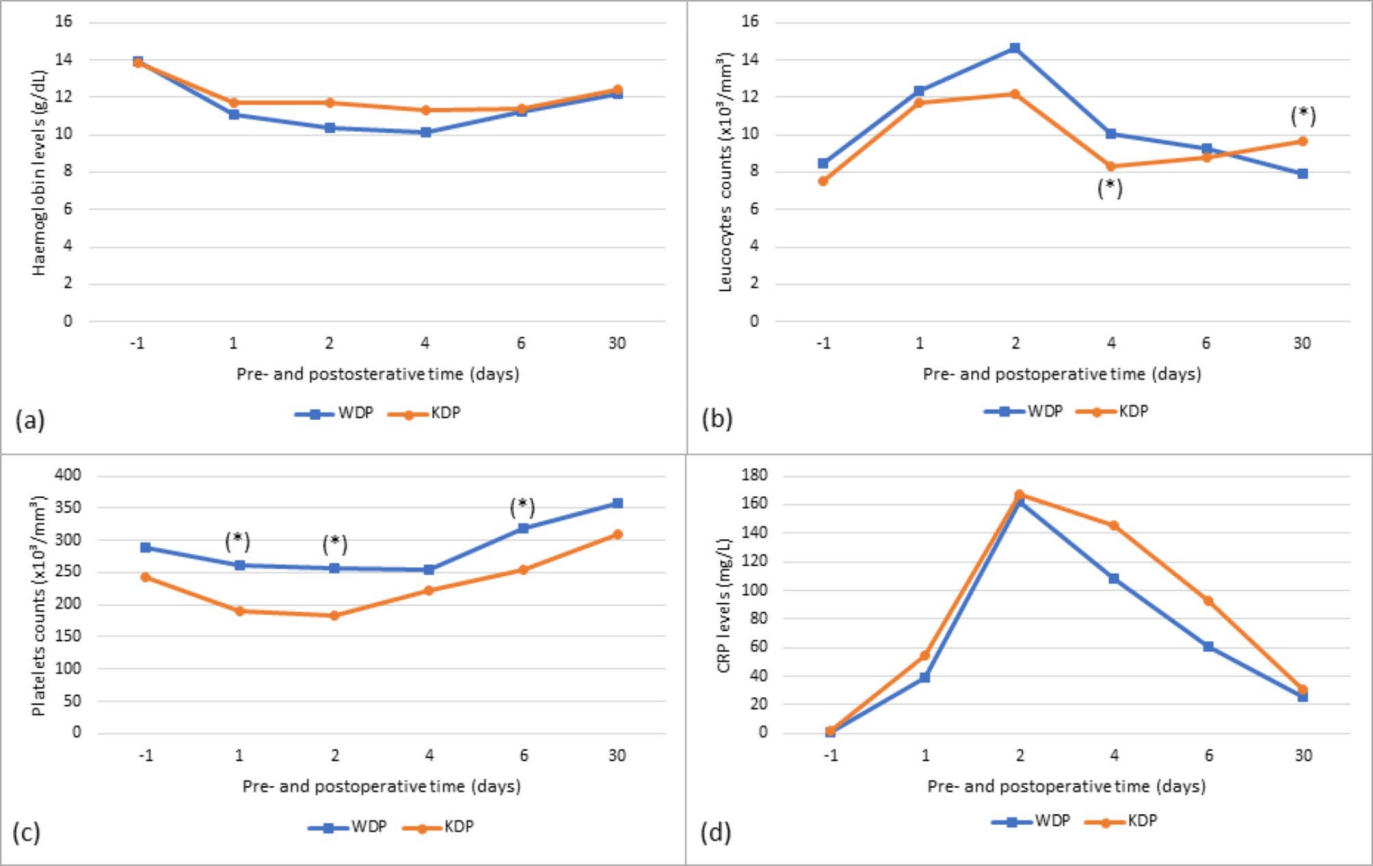
Evolution of biological parameters the day before surgery (-1) and at postoperative days 1, 2, 4, 6 and 30 in the WDP and KDP groups: haemoglobin levels (**a**), leucocyte counts (**b**), platelet counts (**c**), and CRP levels (**d**). A significant difference between groups (p < 0.050) has been marked with a (*) WDP, Warshaw distal pancreatectomy; KDP, Kimura distal pancreatectomy; CRP, C-reactive protein

In both groups, a decrease in mean haemoglobin and platelet levels was observed during the first 2 days post-surgery, which was stabilized (for haemoglobin) or resolved (for platelets) after 4 days (Fig. 1). Conversely, the mean leucocyte and CRP levels increased in both groups until POD 2, and then progressively decreased during the first week until POD 30. Platelet levels at POD 1, POD 2 and POD 6 were significantly higher in the WDP group, as well as leucocytes count at POD 4 compared to KDP group. Conversely, leucocytes count at POD 30 was lower in the WDP group, compared to KDP group.

### Risk factors for partial splenic atrophy

At univariate analysis, prognostic factors for developing splenic atrophy were age, Charlson Comorbidity Index, platelet levels at POD 6, and early splenic infarction (Table 4). None of these factors were independent prognostic factors of splenic atrophy at multivariate analysis.

**Table 4:** Prognostic factors of splenic atrophy

	Univariate analysis			Multivariate analysis		
	OR	95% CI	p	OR	95% CI	p
Male gender (vs. female)	1.067	0.268–4.253	0.927			
Age	1.065	1.002–1.133	0.043	1.055	0.943–1.181	0.350
Body mass index	1.058	0.917–1.221	0.440			
Charlson Comorbidity Index	1.354	1.007–1.821	0.045	1.237	0.719–2.126	0.442
Malignant indication (vs. benign)	0.793	0.205–3.075	0.737			
Operative time	1.000	0.990–1.011	0.962			
Blood loss	1.000	0.997–1.002	0.873			
CCI	0.951	0.889–1.018	0.151			
Haemoglobin level at POD6	0.842	0.550–1.288	0.427			
Leucocyte level at POD6	1.089	0.836–1.418	0.529			
Platelet level at POD6	1.012	1.002–1.021	0.018	1.006	0.985–1.026	0.592
CRP level at POD6	1.000	0.989–1.012	0.943			
Early splenic infarction	15.167	1.674–137.443	0.004			

CCI, Complication Comprehensive Index; CRP, C-reactive protein; POD, postoperative day

## Discussion

This study found that SP-DP with splenic vessels resection resulted in postoperative partial splenic atrophy in almost 30% of cases, but without any clinical impact and no need for secondary splenectomy. Partial splenic volume recovery occurred even in cases of severe splenic ischemia, and no complete splenic atrophy was observed at late imaging. In cases of spleen-preserving distal pancreatectomy with splenic vessels preservation, grade 1 splenic ischemia could be observed following the surgery, without any splenic atrophy at late imaging. Age, Charlson Comorbidity Index and platelets levels at POD 6, and early splenic infarction were prognostic factors for development of splenic atrophy at univariate analysis. To our knowledge, no previous studies have looked for a potential link between postoperative splenic ischemia and splenic volume at late imaging.

Spleen-preserving distal pancreatectomy with splenic vessels resection has been reported to sufficiently preserve splenic vascularization thanks to short gastric vessels, with a low rate of secondary splenectomy of 2-5% due to spleen necrosis [2]. The occurrence of postoperative splenic infarction after SP-DP has been well described, but only a few authors have studied the severity (and the area) of splenic infarction, and none have looked for a possible link between early splenic ischemia and late splenic volume [15–17]. In this study, no patients required splenectomy, even in cases of severe splenic ischemia. With a conservative and observational strategy based on clinical and biological monitoring in the early postoperative period, these patients recovered with a spleen volume greater than 40% after a minimum of 6 months. Yohanathan et al. observed splenic enhancement resolution even after 3 months, with clinically significant sequelae (including infectious) or need for splenectomy at short- and long-term follow-up [18]. Due to the fact that splenic function can be decreased during the period of splenic ischemia, prophylactic vaccination for asplenic/hyposplenic patients could be considered at this time, but not necessarily in the long-term given the extent of spleen volume recovery. There is no consensus about the required minimal volume of the functional splenic tissue. After partial splenectomy, studies support the idea that preservation of 25–30% of splenic volume allows for normal immune function [19]. In cases of splenic ischemia, given the capacity of splenic recovery and regeneration, this percentage of ischemic parenchyma could be higher, as shown in the present study.

Regarding postoperative outcomes, no significant differences were observed between the two techniques of SP-DP regarding the rate of severe complications and complications specifically related to pancreatic surgery. These results are in accordance with the literature [15, 20]. A peripancreatic collection on the transection margin appeared in more than half of the patients, without the need for drainage in three quarters of them. In the last few years, we have adopted a less aggressive approach for these peripancreatic collections. Drainage (transgastric, retrograde, or percutaneous) is now reserved for very symptomatic patients. The rate of development of perigastric varices was similar between the two groups, at approximately 65% of patients, which is surprising in the KDP group with splenic vessels preservation. This could be due to a potentially stenosing haemostasis on the splenic vein during SP-DP, or a local inflammatory state with or without peripancreatic collection extrinsically compressing the splenic vein, although splenic vessels were patent on imaging. However, perigastric vessels were all asymptomatic without any episodes of intraluminal haemorrhage, as also reported in the literature [15]. The choice between the two procedures (WDP or KDP) is normally based on whether the lesion is benign, premalignant, or malignant, depending on the need for lymph node dissection. In view of these similar postoperative results and because preservation of the splenic vessels can be technically more challenging, one should not hesitate to convert to the Warshaw technique.

To our knowledge, no previous study has evaluated the predictive factors of splenic atrophy after SP-DP. Although no factors were significant at multivariate analysis, prognostic factors significant at univariate analysis were identified including age, Charlson Comorbidity Index, platelet levels at POD 6, and early splenic infarction. Regarding the comorbidities considered in the Charlson Comorbidity Index, an elevated index in patients undergoing pancreatic surgery is most frequently due to advanced age, diabetes or cardiovascular disease, comorbidities that can alter healing and splenic revascularisation via the short gastric vessels in WDP. Interestingly, a high platelet level at POD 6, probably related to a relative hyposplenism, predicted the occurrence of splenic atrophy and could be of value to study further in larger studies. Finally, the splenic infarction observed postoperatively in half of the WDP patients is, unsurprisingly, a relevant prognostic factor of splenic atrophy due to splenic hypoperfusion, and could be really interesting as a way to more deeply study the mechanisms of splenic regeneration.

Some limitations of this study must be addressed. This was a retrospective analysis and the investigations used for splenic function assessment were limited and unspecific. We used contrasted-enhanced CT and MRI, as well as postoperative blood cell counts, while 99^m^Tc-labelled, heat-altered, autologous erythrocyte scintigraphy with multimodality single photon emission computed tomography (analysing both function and anatomy) and other haematological and immunological parameters are more specific and reliable, but these modalities are not always available at all sites [21]. Nevertheless, there is a good correlation between the volume of functional splenic tissue and splenic function, as shown in previous studies, and CT is easily accessible in the perioperative period and allows the evaluation of other postoperative outcomes [22, 23].

## Conclusions

In conclusion, ischemia of splenic parenchyma appeared in one-half of patients undergoing SP-DP with splenic vessels removal at early postoperative imaging, but with a good recovery of splenic volume at late postoperative imaging. Almost 30% of patients developed partial splenic atrophy but without clinical impact; no complete splenic atrophy was observed. Age, Charlson Comorbidity Index, platelet levels at POD 6, and early splenic infarction were prognostic factors for development of splenic atrophy at univariate analysis. No differences were observed in postoperative outcomes after SP-DP with splenic vessels removal versus preservation. In view of these results on splenic outcomes, one should not hesitate to convert to the Warshaw technique.

## Data Availability

The datasets used and analysed during the study are available from the corresponding author upon reasonable request.

## Abbreviations

CCI: Comprehensive Complication Index
CRP: C-reactive protein
CT: Computed tomography
DP: Distal pancreatectomy
KDP: Kimura spleen-preserving distal pancreatectomy
MRI: Magnetic resonance imaging
POD: Postoperative day
SP-DP: Spleen-preserving distal pancreatectomy
SVR: Spleen volume ratio
WDP: Warshaw spleen-preserving distal pancreatectomy
